# Phylogenetic Relatedness of Circulating HIV-1C Variants in Mochudi, Botswana

**DOI:** 10.1371/journal.pone.0080589

**Published:** 2013-12-11

**Authors:** Vladimir Novitsky, Hermann Bussmann, Andrew Logan, Sikhulile Moyo, Erik van Widenfelt, Lillian Okui, Mompati Mmalane, Jeannie Baca, Lauren Buck, Eleanor Phillips, David Tim, Mary Fran McLane, Quanhong Lei, Rui Wang, Joseph Makhema, Shahin Lockman, Victor DeGruttola, M. Essex

**Affiliations:** 1 Department of Immunology and Infectious Diseases, Harvard School of Public Health, Boston, Massachusetts, United States of America; 2 Botswana–Harvard AIDS Institute Partnership, Gaborone, Botswana; 3 Department of Biostatistics, Harvard School of Public Health, Boston, Massachusetts, United States of America; Vanderbilt University, United States of America

## Abstract

**Background:**

Determining patterns of HIV transmission is increasingly important for the most efficient use of modern prevention interventions. HIV phylogeny can provide a better understanding of the mechanisms underlying HIV transmission networks in communities.

**Methods:**

To reconstruct the structure and dynamics of a local HIV/AIDS epidemic, the phylogenetic relatedness of HIV-1 subtype C *env* sequences obtained from 785 HIV-infected community residents in the northeastern sector of Mochudi, Botswana, during 2010–2013 was estimated. The genotyping coverage was estimated at 44%. Clusters were defined based on relatedness of HIV-1C *env* sequences and bootstrap support of splits.

**Results:**

The overall proportion of clustered HIV-1C *env* sequences was 19.1% (95% CI 17.5% to 20.8%). The proportion of clustered sequences from Mochudi was significantly higher than the proportion of non-Mochudi sequences that clustered, 27.0% vs. 14.7% (p = 5.8E-12; Fisher exact test). The majority of clustered Mochudi sequences (90.1%; 95% CI 85.1% to 93.6%) were found in the Mochudi-unique clusters. None of the sequences from Mochudi clustered with any of the 1,244 non-Botswana HIV-1C sequences. At least 83 distinct HIV-1C variants, or chains of HIV transmission, in Mochudi were enumerated, and their sequence signatures were reconstructed. Seven of 20 genotyped seroconverters were found in 7 distinct clusters.

**Conclusions:**

The study provides essential characteristics of the HIV transmission network in a community in Botswana, suggests the importance of high sampling coverage, and highlights the need for broad HIV genotyping to determine the spread of community-unique and community-mixed viral variants circulating in local epidemics. The proposed methodology of cluster analysis enumerates circulating HIV variants and can work well for surveillance of HIV transmission networks. HIV genotyping at the community level can help to optimize and balance HIV prevention strategies in trials and combined intervention packages.

## Introduction

The HIV/AIDS epidemic is one of the biggest public health challenges, with the major burden of HIV infections being in southern Africa [1]. Combination antiretroviral therapy (cART) saves millions of lives around the world, and the ART-as-Prevention approach has become a promising part of combination prevention interventions aimed at controlling the HIV/AIDS epidemic. In the reality of changing trends in HIV epidemiology [2], a better understanding of the mechanisms underlying the HIV transmission networks in communities may help to properly balance and maximize the efficiency of public health interventions, such as ART-containing strategies known as Treatment as Prevention (TasP) and Pre-Exposure Prophylaxis (PrEP).

The uncertainty on the location of potential HIV transmission source(s) may lead to lower efficiency in HIV prevention strategies because prevention strategies target specific populations. If an HIV prevention trial does not take into account patterns of HIV transmission across communities, it remains unclear whether HIV transmission(s) could be effectively prevented with a certain strategy. For example, TasP in a single community could prevent HIV transmissions *within* the community, but could not prevent HIV transmissions from any *outside* source(s). However, if the same HIV prevention strategy, e.g., scaled-up TasP, is used broadly in multiple adjacent communities and the potential source of HIV transmission is within one of the targeted communities, the HIV transmission is likely to be prevented. In contrast, PrEP in a single community should be able to prevent HIV transmissions from an *outside* source, but its cost could be substantially higher.

HIV genotyping can be used to reconstruct the structure of viral transmission networks and patterns of HIV spread in communities. To characterize the complexity and heterogeneity of the local epidemic, the circulating HIV variants in communities can be enumerated and their sequence signatures identified. The HIV sequences found in clusters can be associated with viral transmission chains, and the community-unique viral variants can be distinguished from HIV variants spreading across communities. A combination of HIV genotyping with the relevant socio-demographic and behavioral data can provide powerful knowledge on patterns and dynamics of HIV transmission network across communities, which can guide HIV prevention and intervention strategies to identify and target specific populations.

The definition of the term *cluster* is critical for a proper, biologically meaningful interpretation of the data in cluster analysis. In the context of viral transmission, clustering has been used as a tool for identification of transmission chains, as it is believed that clustered viral sequences belong to the same transmission chain. Phylogenetic inference has been successfully used in forensic trials to provide evidence either for [3]–[8] or against [9]–[11] HIV-1 transmission. Phylogeny has been used to prove or reject linkage in discordant couples upon partner's HIV infection in large clinical trials [12], [13]. Cluster analysis has helped to determine the phylodynamic structure of HIV/AIDS epidemics and reveal HIV transmission networks [14]–[30], as well as reconstruct outbreaks [31]–[40]. The majority of previous studies focused on HIV-1 subtype B-infected populations of men who have sex with men (MSM), utilizing partial *pol* sequences for phylogenetic inference, and using relatively stringent criteria/thresholds for cluster definition.

In this study we focus on relatedness of HIV-1 subtype C *env* sequences from Mochudi, Botswana, at the community or population level. We defined the term “circulating HIV variant” as a clustered group of similar viruses representing a chain of HIV transmissions that can be distinguished phylogenetically from other clustered or non-clustered viruses. We associated the identified viral clusters with circulating HIV variants in the community, leaving aside links related to directional HIV transmissions between particular individuals. High sampling coverage in the local HIV epidemic in Mochudi allowed us to decrease the phylogenetic error [41], [42]. Due to the population level of the cluster analysis, high evolutionary rates within HIV-1 *env*, and uncertain time of HIV infection in the majority of sampled individuals from Mochudi, we used relatively relaxed thresholds for cluster definition to avoid elimination of phylogenetic signal present in the dataset. To identify clusters in this study we developed a two-step nested cluster analysis using the Maximum Likelihood (ML) plus bootstrap approach.

The phylogenetic relatedness of HIV-1C *env* sequences from Mochudi was estimated in the context of other HIV-1C *env* sequences available in the public domain. The study aimed to address proportions of clustered sequences within different subsets, enumerate and characterize HIV variants circulating in Mochudi, assess potential associations between clusters and HIV-1 RNA load, and evaluate clustering patterns among Mochudi seroconverters.

## Materials and Methods

### Ethics statement

This study was conducted according to the principles expressed in the Declaration of Helsinki, and was approved by the Health Research and Development Committee (HRDC) of the Republic of Botswana, and the Office of Human Research Administration (OHRA) of the Harvard School of Public Health. All adult study subjects provided written informed consent for participation in the study; all minor study subjects provided written informed assent, and each minor's guardian provided written informed consent, for their participation in the study.

### Study subjects from Mochudi, Botswana

Mochudi is a village in Botswana with a population of 44,339 people in 2011. Mochudi was settled in 1871, and is located in the Kgatleng District about 37 km (23 mi) northeast of the national capital, Gaborone. Geographically Mochudi is divided into three sectors by a river and small hills. The results presented in this study are part of the NIH-funded Methods for Prevention Packages Program (MP3) study *An HIV Prevention Program for Mochudi, Botswana* (R01 AI083036; working name of the study: Mochudi Prevention Project, or MPP; PI: M. Essex). One of the key components of the MPP was enhanced HIV testing and counseling (HTC) in households among 16–64-year-old residents. Estimates of HIV-1 incidence and prevalence over time were the major goals of the enhanced HTC campaign in the northeastern sector of Mochudi (estimated total population of about 15,000) performed from May 2010 to May 2013. The baseline and two follow-up enhanced HTC campaigns targeted residents of the Mochudi community in households. The community mobilization and engagement, as well as procedures of consenting, HIV testing, counseling, collection of data and sampling, were the same in all enhanced HTC campaigns. During the household visit, the study recruiters approached the head of household, identified eligible household members, presented the MPP study and offered HIV testing and counseling. Eligible residents were asked to complete an individual questionnaire with socio-demographic and HIV-related information including ART status and patterns of sexual behavior (e.g., whether sexual partner resides outside of Mochudi), and to donate a blood sample for rapid HIV test and viral genotyping (if HIV positive). The capillary blood samples were collected as dry blood spots (DBS) using a single DBS card that was labeled, dried and transported to the lab. Testing for anti-HIV-1 antibodies was performed in households using National HIV Testing guidelines and Botswana-government–approved two rapid tests. The HIV testing algorithm included Determine HIV-1/2 Rapid Test (Abbott Diagnostic Division, Belgium/Luxemburg) and Uni-gold™ HIV rapid test (Trinity Biotech, Wicklow, Ireland) in parallel. Only concordant results in two tests were considered valid. If results were discordant, subject was invited to a clinic and blood specimen was collected by phlebotomy and transported to a certified reference laboratory using cool boxes within 4 hours from sampling, and processed within 8 hours. The confirmatory HIV testing was performed using EIA (Murex HIV 1.2.0 test - Murex Biotech Ltd, Dartford, Kent, England, or Vironostika Uniform II plus O EIA - BioMerieux Inc, Durham, NC) and/or Western blot (Genetic Systems HIV-1 Western Blot, Bio-Rad Laboratories, Redmond, WA). The results of EIA and/or Western Blot superseded the discordant or indeterminate results obtained in the field. All HIV tests were conducted according to the manufacturer's guidelines. ART-naïve HIV-infected individuals were invited to a clinic to determine their eligibility for initiation of ARV treatment that included collection of venous blood by phlebotomy for CD4 and HIV-1 RNA testing. CD4 counts were enumerated by standard flow cytometry using a 4-color FACSCalibur and the TriTest kit (BD Biosciences, San Diego, CA). The HIV-1 RNA load was quantified in DBS or in plasma (if venous blood was collected in the clinic) by Roche Ampliprep/Amplicor v.1.5 or Abbott m2000sp/Abbott m2000rt. Individuals reported to be on ART received treatment through the National ART program and initiated ART based on Botswana national guidelines (treatment of all adults with CD4≤350 cells/µL or WHO Stage III/IV). Individuals on ART were not invited to a clinic and their CD4 was not tested in this study. To identify seroconverters, the follow-up enhanced HTC campaigns targeted individuals who had tested HIV-negative in a previous campaign. Seroconverters were identified based on a paired HIV-negative and HIV-positive tests.

All data presented in the study refer to the northeastern sector of Mochudi.

The proportion of people who were HIV-1 positive in the Mochudi sample among 16–64-year-old individuals was 20.6% (95% CI 19.6% to 21.7%). A total of 6,165 age-eligible residents in the northeastern sector of Mochudi were tested for HIV-1 status, and 1,272 of them were found to be HIV positive. Among these HIV-positive individuals, 75.2% were female. The number of HIV-positive individuals within the age range 16–64 years old in the northeastern sector of Mochudi was estimated at 1,792 (15,000×0.58×0.206, where 0.58 is the proportion of age-eligible individuals), which translates to a sampling coverage of about 71% (95% CI 69% to 73%; 1,272 of 1, 792) of the estimated number of age-eligible HIV-infected individuals, and 44% (95% CI 41% to 46%; 785 of 1,792, where 785 is the number of generated viral sequences from samples collected in Mochudi) HIV genotyping coverage. During the survey, a total of 27 seroconverters were identified.

### Additional HIV-1C env sequences from Botswana

New HIV-1C *env* sequences were generated within the GWAS study in Botswana (RC4 AI092715; PI: M. Essex) which aims to analyze viral diversity and evolutionary dynamics of HIV-positive individuals. The set included in the current study was comprised of 100 HIV-1 *env* sequences obtained from a subset of the HIV-infected women who participated in the earlier Mashi study (R01 HD37793; PI: M. Essex) [43], [44] and who signed a consent form, and donated a blood sample for genotyping. Sample collection was performed at Princess Marina Hospital, located in the national capital, Gaborone.

### HIV-1C env sequences retrieved from the public domain

**Figure 1:**
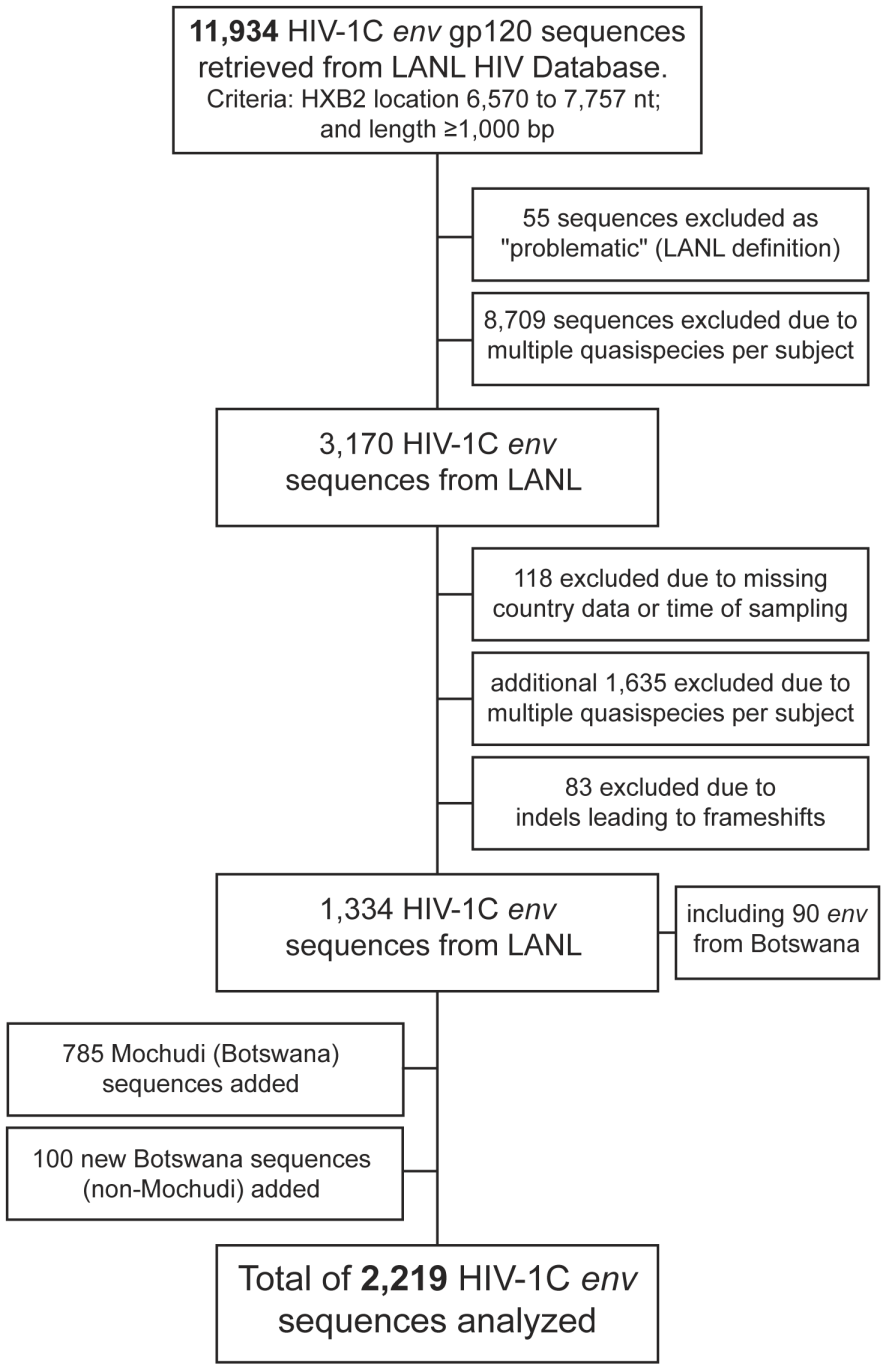
HIV-1C *env* sequences included in analysis. A total of 11,934 non-recombinant HIV-1C *env* sequences spanning at least 1,000 bp within the gp120 V1–C5 region were selected from the LANL HIV Database. After initial on-line filtering, 3,170 sequences were selected. The applied manual filtering left 1,334 sequences. Together with 885 new Botswana sequences including 785 sequences known to be from Mochudi, a total of 2,219 HIV-1C *env* sequences were included in analysis.

A total of 11,934 HIV-1C non-recombinant *env* sequences spanning the V1-C5 region of gp120 (HXB2 location 6,570 to 7,757 nt) with length of at least 1,000 bp were retrieved from the Los Alamos National Laboratory HIV Database (http://www.hiv.lanl.gov/content/sequence/HIV/mainpage.html) on February 25, 2013 (Figure 1). After exclusion of problematic sequences and multiple quasispecies from the same subjects through the online auto filter at the HIV Database, the total number of available sequences was reduced to 3,170. The results of further filtering of HIV-1C *env* sequences from the public domain performed manually are presented in Figure 1 and in the Results section below (*HIV-1C env sequences included in analysis* subsection). The country-specific sampling coverage was estimated as a ratio of the number of HIV-1 *env* sequences analyzed per country to the UNAIDS-estimated number of HIV-positive individuals in each country (http://www.unaids.org/en/regionscountries/countries/).

The following groups and subsets were used to address different questions in the study:

(1) Mochudi sequences, n = 785; (2) Botswana non-Mochudi sequences, n = 190; (3) Botswana sequences, n = 975 (785+190); (4) non-Botswana sequences, n = 1,244; and (5) non-Mochudi sequences, n = 1,434 (190+1,244). We also defined HIV variants circulating in a single community as *community-unique*, e.g., circulating Mochudi-unique variants, and HIV variants spreading across multiple communities as *community-mixed*, e.g., circulating Mochudi-mixed variants.

### Nucleic acid extraction, amplification and sequencing

Blood samples from participants in the MPP study were collected either as DBS in households, or as standard venous blood collected in clinic. DBS and processed buffy coats were stored at minus 70**°**C immediately after delivery to the lab and processing, and shipped for HIV genotyping to the Harvard School of Public Health (HSPH) lab in Boston, MA on dry ice using World Courier, a commercial shipping service. Nucleic acid from DBS was extracted by the EZ1 DNA Tissue Kit (cat # 953034, Qiagen) and from buffy coats by the EZ1 DNA Blood 200 µl kit (cat # 951034, Qiagen) using the automated EZ1 Advanced system (Qiagen, Germantown, MD) according to the manufacturer's instructions. Proviral DNA was used as a template for amplification and sequencing. The methodology of the HIV-1C *env* gp120 V1–C5 amplification has been presented in detail elsewhere [45]–[47]. Briefly, the analyzed region in HIV-1C *env* corresponded to nucleotide positions 6,570 to 7,757 of HXB2. The FastStart High Fidelity Enzyme Blend (Roche Diagnostics, Indianapolis, IN) was used for all PCR amplifications with the set of ED3/ED5/ED12/ED14 primers [48]. Amplicons were purified by Exo-SAP [49], and were sequenced directly at both strands on the ABI 3730 DNA Analyzer using BigDye technology. Sequence contigs were assembled by SeqScape v.2.7. HIV genotyping is still ongoing in the MPP study. The current study is based on analysis of HIV-1C sequences from 785 individuals from Mochudi that were generated by May 9, 2013.

### Multiple sequence alignment

The initial codon-based multiple sequence alignment was performed by Muscle [50] with gap penalty of −3.2 and gap extension of −0.8 followed by minor manual adjustments in BioEdit [51]. All variable loops in gp120 except V3 were re-aligned with low penalties for gap opening and gap extension. Nucleotide ClustalW [52] with running parameters set to 5 for gap opening and 2 for gap extension in pairwise alignment and 0 for both gap opening and gap extension in multiple alignment, and 0.25 as a transition weight, was used as the primary re-alignment algorithm. Codon-based re-alignment by Muscle with running parameters set to −0.8 for gap opening and −0.4 for gap extension, and re-alignment with gradual gap stripping, was also used to address whether codon-based re-alignment, or positions with gaps, affect clustering. We found little to no effect of codon-based re-alignment or gap stripping on clustering patterns, and thus only clustering results based on nucleotide re-alignment are presented in this study. However, the results of cluster-specific signatures are based on translated amino acids that required codon alignment throughout the analyzed region of HIV-1C *env*.

### Flow of cluster analysis

**Figure 2:**
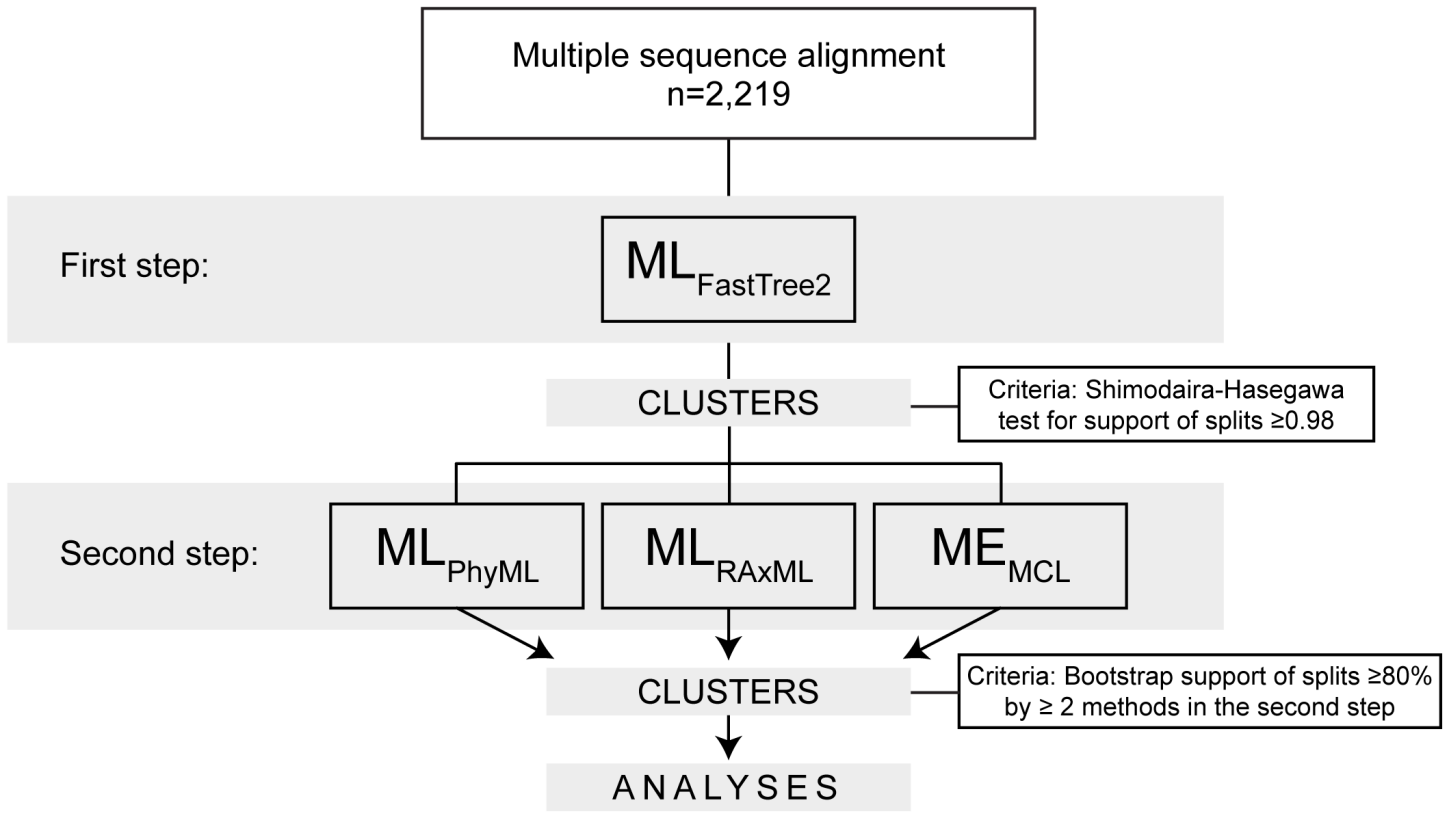
Flow of cluster analysis. A total of 2,219 HIV-1C *env* sequences were included. After re-alignment of variable loops, the first step ML analysis was implemented by FastTree2, which selected 969 sequences based on the Shimodira-Hasegawa test for splits support ≥0.98. Two ML analyses implemented by PhyML and RAxML, and ME analysis using MCL model, were performed in the second step. Clusters were identified based on bootstrap support of ≥80% in at least 2 of 3 methods in the second step.

The phylogenetic relatedness among HIV-1C *env* sequences was estimated by applying a two-step nested cluster analysis (Figure 2). Generally, the nonparametric bootstrap has been widely used in phylogenetic analysis to assess the clade support of phylogenetic trees. In this study, as the first step of cluster search, the ML [53] analysis was implemented by FastTree2 (ML_FastTree2_) [54], [55]. Viral sequences with the splits support of ≥0.98 were selected for the next step. Two alternative ML methods implemented by PhyML (ML_PhyML_) [56] and RAxML (ML_RAxML_) [57], and Minimum Evolution (ME) [58] implemented by MEGA5 [59] using the Maximum Composite Likelihood method [60] (ME_MCL_), were applied to the subset of clustered sequences identified in the first step (Figure 2). The ML_PhyML_, ML_RAxML_, and ME_MCL_ in the second step were used with 100 bootstrap replicates for each set of *env* sequences. The web-based server T-rex [61] was utilized for bootstrapping in the ML_PhyML_ analysis. In the bootstrap analyses by ML_PhyML_, some large data sets were terminated before runs of 100 replicates were completed. In these cases, runs were repeated until at least 100 replicates were reached, and the consensus support of splits was estimated by the SumTrees v.3.3.1 package with DendroPy 3.8.0 [62]. The RAxML runs were performed using the high-performance computing cluster Odyssey (http://rc.fas.harvard.edu/kb/high-performance-computing/architectural-description-of-the-odyssey-cluster/) at the Faculty of Arts and Sciences, Harvard University (https://rc.fas.harvard.edu/). In the second step of cluster analysis, bootstrap support of splits ≥80% was used as a cut-off value. Viral sequences confirmed by at least 2 of the 3 methods of phylogenetic inference in the second step were considered to be in clusters.

### Statistical analysis

The statistical analysis was performed in R. The proportions were estimated with 95% confidence intervals (95% CI). Comparisons of continuous outcomes between two groups were performed using the Wilcoxon Rank Sum test. P-values less than 0.05 were considered statistically significant. All reported p-values are 2-sided.

### Graphics

The inferred phylogenetic trees were visualized using SeaView [63] and FigTree v.1.4 (http://tree.bio.ed.ac.uk/software/figtree/). Bar plots for the cluster size distribution and boxplots for comparison of HIV-1 RNA between groups were produced in R. All images were finalized in Adobe Illustrator CS6.

### Accession numbers

The accession numbers of HIV-1C env sequences used in the study from the public domain are presented in Table S1. The accession numbers of the 785 Mochudi sequences are KF373894–KF374678. The accession numbers of the 100 viral sequences from the GWAS study are KF373794–KF373893.

## Results

### Questions addressed by the study

To better understand clustering patterns among circulating HIV-1 C variants in the community and identify clusters of viral sequences, the phylogenetic relatedness among HIV-1C *env* sequences originating from the village of Mochudi, Botswana, was estimated. The newly generated set of 785 HIV-1C *env* sequences from Mochudi was complemented by HIV-1C *env* sequences available in the public domain. Clusters were defined based on bootstrap support of splits ≥80% in at least 2 of the 3 methods of phylogenetic inference. The current study addressed the following questions: (1) Proportions of clustered HIV-1C *env* sequences overall, and within the Mochudi subset; (2) Cluster size distribution; (3) Proportion of Mochudi-unique clusters vs. mixed clusters; (4) Enumeration of HIV variants circulating in Mochudi and identification of their sequence signatures; (5) Potential association between clustering and HIV-1 RNA load; and (6) Clustering of Mochudi seroconverters.

### HIV-1C env sequences included in analysis

The initial number of non-recombinant HIV-1C *env* sequences available in the public domain was 11,934 but was reduced to 3,170 after initial online filtering. The data set was further refined by excluding viral sequences with missing origin or time of sampling, or multiple indels leading to frameshifts. In addition, 1,635 sequences were excluded due to the presence of multiple quasispecies identified manually including multiple submissions of sequences from the same individuals. Mother-infant pairs from PMTCT studies (primarily from Malawi) were not excluded from analysis. The resulting set of 1,334 HIV-1C *env* sequences retrieved from the public domain was complemented by 785 new sequences from Mochudi, Botswana, and 100 newly generated HIV-1C *env* sequences collected in Gaborone (see Materials and Methods).

The final set included 2,219 HIV-1C *env* sequences from 38 countries. Table 1 shows per-country representation of HIV-1C *env* sequences included in analysis. The majority of HIV-1C sequences (93.7%) were derived from six countries including 975 from Botswana (785 known to be from Mochudi), 674 from South Africa, 179 from Malawi, 109 from Zambia, 81 from India, and 62 from Tanzania. Fewer than 20 HIV-1C *env* sequences per country were available in the public domain from the remaining 32 countries.

**Table 1:** Selected countries representation of HIV-1C *env* sequences included in analysis.

Country code	Total sequences		Years of sampling
	N	%	
BW, Botswana	975	43.9%	1996–2012
IN, India	81	3.7%	1991–2008
MW, Malawi	179	8.1%	1989–2010
TZ, Tanzania	62	2.8%	1997–2008
ZA, South Africa	674	30.4%	1981–2010
ZM, Zambia	109	4.9%	1988–2007
Other countries (n = 32) with fewer than 20 sequences per country	139	6.3%	1987–2010
Total:	2,219	100%	

### Recombination analysis

Before the phylogenetic analysis was performed, the entire set of 2,219 *env* sequences was tested for the presence of recombination signal. The Phi Test [64] implemented by the Splits Tree4 [65], [66] did not find statistically significant evidence for recombination in the analyzed data set (p = 1.0). All Botswana sequences were also analyzed for recombination signal using RDP4 [67], a software package for statistical identification and characterization of recombination events that utilizes non-parametric recombination detection methods, such as RDP [68], GENECONV [69], Bootscan/Recscan [70], MaxChi [71], Chimaera [72], SiScan [73] and 3Seq [74]. RDP4 does not require reference sequences, which makes analysis of viral quasispecies from epidemiologically unlinked patients more practical [46]. A total of five sequences from Botswana including 3 from Mochudi demonstrated evidence for recombination signal in RDP4 by 2 to 4 out of 9 methods of analysis (Table S2). All recombination points were unique.

### Proportion of clustered HIV-1C env sequences

Applying the two-step nested approach for cluster identification to the entire set, 423 viral sequences were found in clusters corresponding to 19.1% (95% CI 17.5% to 20.8%) of all analyzed HIV-1 *env* sequences. However the proportion of clustered sequences originating from Mochudi was significantly higher than the proportion of non-Mochudi sequences, 27.0% vs. 14.7; p = 5.8E-12 (Fisher exact test); p = 3.0E-12 (2-sample test for equality of proportions with continuity correction; Table 2). None of the sequences with recombination signal were found within clusters.

**Table 2:** Proportion of clustered HIV-1C *env* sequences.

Strict cluster analysis	Clustered sequences, n	Total sequences, n	Proportion of clustered sequences	95% CI, from	95% CI, to	Fisher exact test, p-value	2-sample test for equality of proportions, p-value	95% CI of the difference, from	95% CI of the difference, to
Mochudi sequences	212	785	0.2701	0.2396	0.3028				
Non-Mochudi sequences	211	1,434	0.1471	0.1294	0.1668	5.76E-12	3.03E-12	0.1280	0.2355

### Cluster size distribution

**Figure 3:**
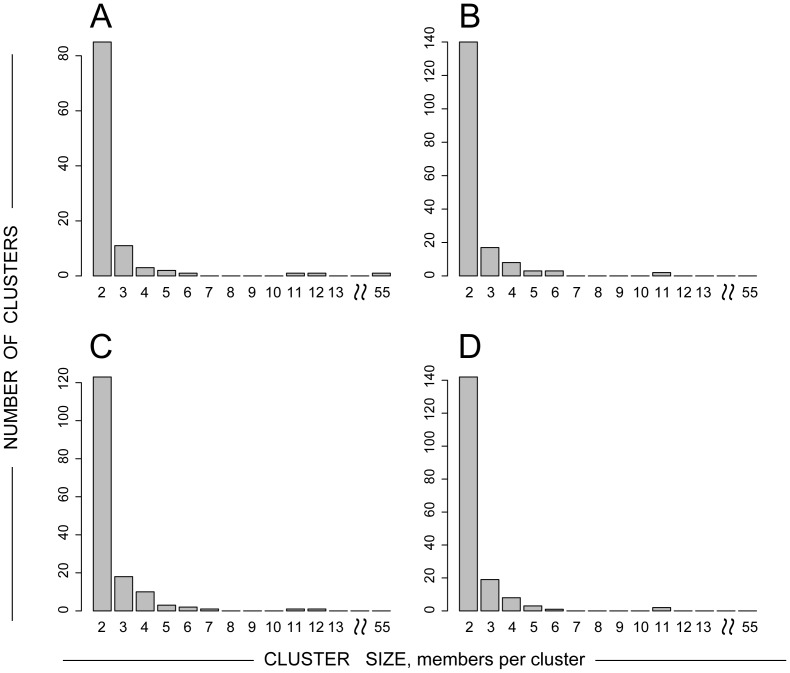
The uniform cluster size distribution followed the power law. Axis *x* shows cluster size as the number of members per cluster. Axis *y* shows the number of identified clusters. **A:** ML_FastTree2_. **B:** ML_FastTree2_ + ML_PhyML_. **C:** ML_FastTree2_ + ML_RAxML_. **D:** ML_FastTree2_ + ME_MCL_. Note: The cluster with 55 members was identified in the ML_FastTree2_ analysis only, and was not confirmed in the second-step analysis by the ML or ME phylogeny.

The size of clusters ranged from 2 to 11 sequences per cluster. However the majority of identified clusters were dyads: 144 of 178 (80.9%) in PhyML analysis, 140 of 185 (75.7%) in RAxML analysis, and 142 of 175 (81.1%) in NJ-MCL analysis. The histogram of identified HIV-1C *env* clusters sizes (Figure 3) has a long tail resembling a power law distribution [75], within all analytical methods. There were 34 (19.1%; 95% CI 13.8% to 25.8%; PhyML analysis) clusters with 3+ members. The proportion of HIV-1C *env* sequences in clusters with 3+ members was 6.4% (141 of 2,219; 95% CI 5.4% to 7.5%; PhyML analysis) of the total number of analyzed sequences. The proportion of Mochudi sequences within clusters with 3+ members was significantly higher than the proportion of non-Mochudi sequences, 12.1% vs. 3.0%; p<2.2E-16 (Table 3).

**Table 3:** Proportion of clustered HIV-1C *env* sequences in clusters with 3+ members.

Strict cluster analysis, 3+ clusters	Clustered sequences, median (IQR)	Total sequences, n	Proportion of clustered sequences	95% CI, from	95% CI, to	Fisher exact test, p-value	95% CI of the difference, from	95% CI of the difference, to	2-sample test for equality of proportions, p-value
Mochudi sequences	95 (95–113)	785	0.1210	0.0994	0.1464				
Non-Mochudi sequences	43 (40–50)	1,434	0.0300	0.0220	0.0405	<2.2E-16	0.2731	0.4406	<2.2E-16

### Clustered Mochudi sequences

To assess cluster structure by origin of sampling, we analyzed the composition of identified clusters. Specifically, the four types of clusters included (1) Mochudi-unique, containing only sequences from Mochudi, (2) mixed, containing both Mochudi and non-Mochudi sequences, (3) non-Mochudi clusters from Botswana that included no Mochudi sequences, and (4) non-Botswana clusters. The analysis was performed with the entire set of viral sequences and also for a subset of clusters with 3+ members.

**Figure 4:**
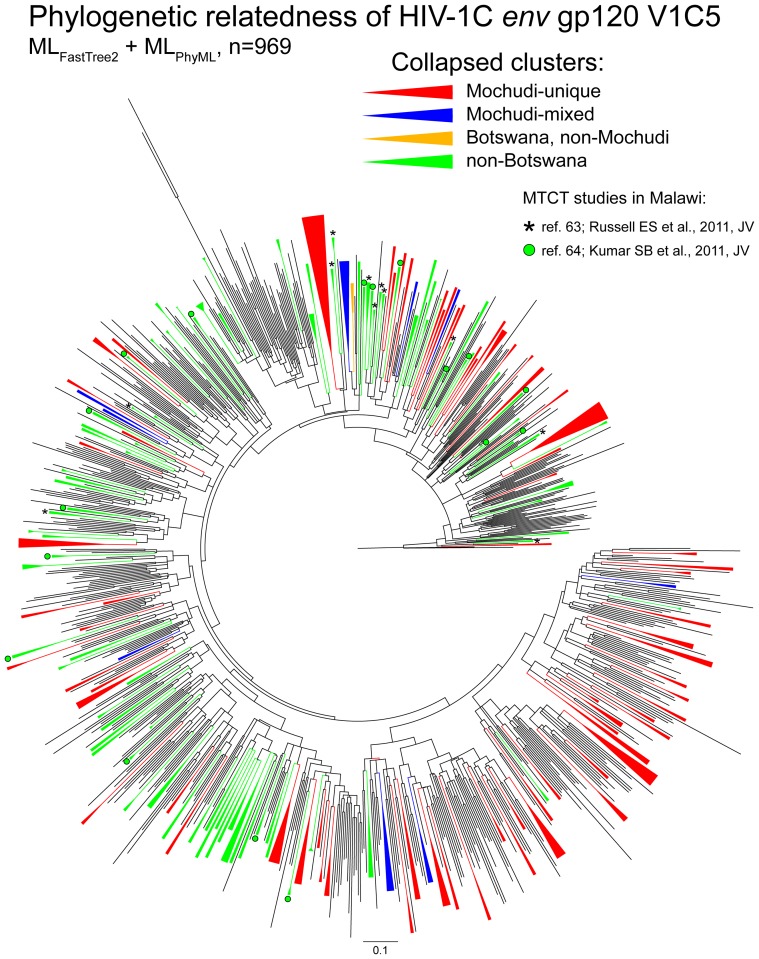
Phylogenetic relatedness of HIV-1C *env* sequences based on the ML_FastTree2_ + ML_PhyML_ analysis. The identified clusters with bootstrap support of ≥80% are collapsed. The collapsed clusters shown in red represent Mochudi-unique clusters; in blue, Mochudi mixed clusters with other Botswana sequences; in orange, non-Mochudi clusters with Botswana sequences; and in green, non-Botswana clusters. Clusters with mother-infant pairs from two MTCT studies in Malawi are shown by asterisks [78] and green circle [79].

A total of 212 (27.0%; 95% CI 24.0% to 30.3%) HIV-1C *env* sequences from Mochudi were found in 83 clusters (Figure 4). The majority of the identified clusters, 73 (88.0%; 95% CI 78.5% to 93.8%), were Mochudi-unique, and contained 191 (90.1%; 95% CI 85.1% to 93.6%) Mochudi sequences. The remaining 21 clustered sequences from Mochudi were found in 10 mixed clusters together with 11 sequences from other parts of Botswana. In this study, none of the Mochudi sequences clustered with HIV-1C *env* sequences from outside of Botswana. Thus, 191 (24.3%; 95% CI 21.4% to 27.5%) of the total number of analyzed Mochudi sequences were Mochudi-unique, and another 21 (2.7%; 95% CI 1.7% to 4.1%) Mochudi sequences were in mixed clusters with other Botswana sequences.

**Figure 5:**
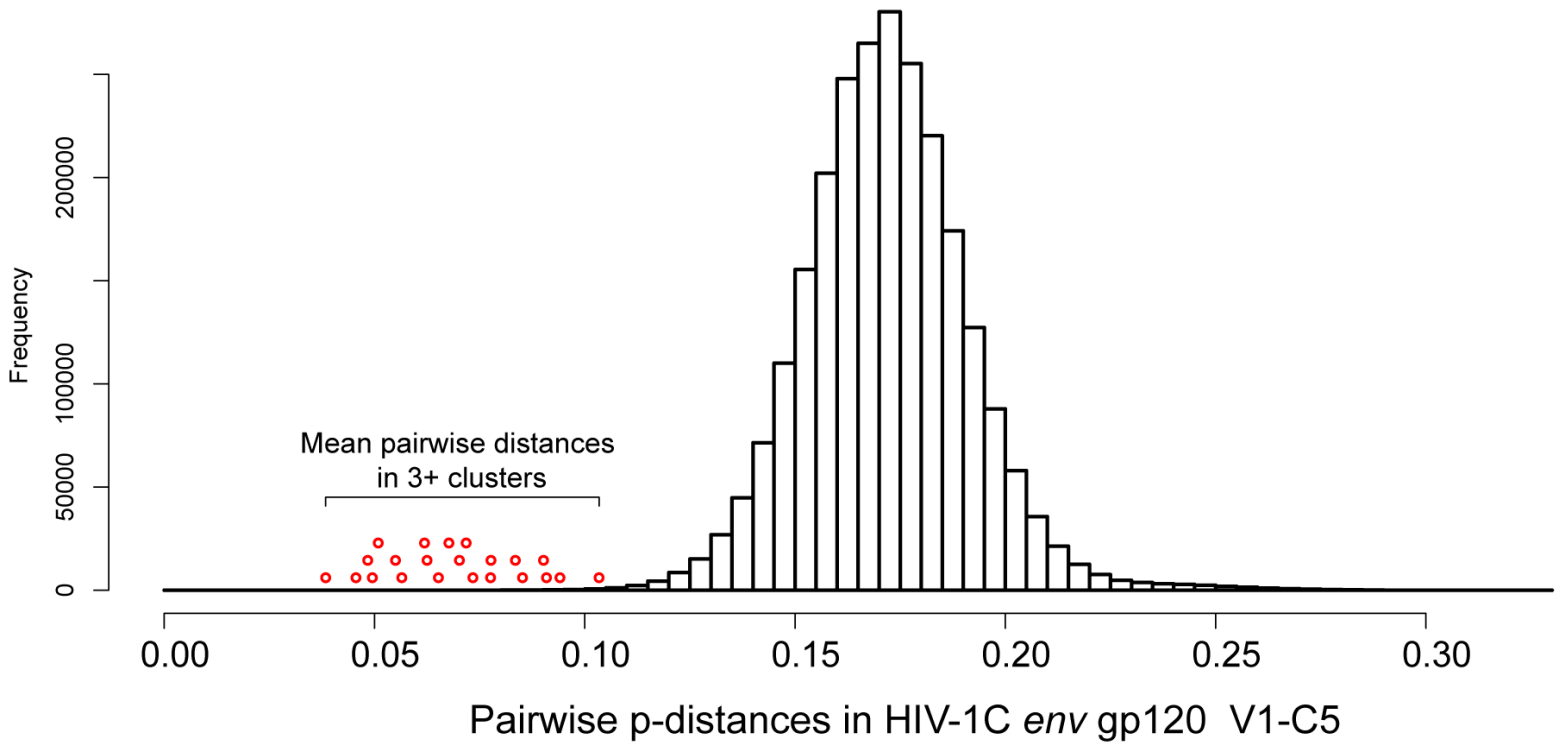
Distribution of pairwise p-distances within the analyzed set of HIV-1C *env* gp120 V1–C5 region, n = 2,219. Mean pairwise p-distances for 22 clusters with 3+ members are denoted as red circles on the left shoulder of overall distribution. The position of mean pairwise distances is only relevant to the p-distances on the *x* axis.

Within the subset of clusters with 3+ members, 81 (10.3%; 95% CI 8.3% to 12.7%) Mochudi sequences were within 18 Mochudi-unique clusters and 19 (2.4%; 95% CI 1.5% to 3.8%) sequences from Mochudi were found in 4 mixed clusters with HIV-1C sequences from other parts of Botswana. The descriptive statistics of pairwise p-distances within 22 clusters with 3+ members that include Mochudi HIV-1C *env* sequences are presented in Table 4. For comparison, the pairwise p-distances for the entire set of analyzed HIV-1C *env* sequences had a median (IQR) of 0.1720 (0.1600 to 0.1840) and mean of 0.1722, and ranged from 0.0020 to 0.3290. The distribution of pairwise p-distances for the entire set of analyzed sequences and mean pairwise distances within clusters with 3+ members (Figure 5) highlights relationships between clustered and non-clustered HIV-1C *env* sequences. Despite relatively high absolute values (particularly in comparison with HIV-1 *pol* datasets), pairwise distances within clusters are substantially lower than pairwise distances in the majority of non-clustered sequences.

**Table 4:** Pairwise p-distances within clusters with 3+ members.

Cluster number	Cluster size	Incident case	Min	1st Quartile	Median	Mean	3rd Quartile	Max
50	4	No	0.0347	0.0464	0.0566	0.0550	0.0672	0.0684
67	3	No	0.0451	0.0472	0.0493	0.0495	0.0517	0.0540
68	3	Yes	0.0731	0.0820	0.0908	0.0852	0.0912	0.0916
71	3	No	0.0587	0.0647	0.0707	0.0677	0.0722	0.0737
84	6	No	0.0434	0.0491	0.0555	0.0565	0.0622	0.0713
85	3	No	0.0190	0.0309	0.0427	0.0384	0.0481	0.0534
94	5	Yes	0.0332	0.0566	0.0610	0.0619	0.0731	0.0792
95	4	No	0.0604	0.0694	0.0719	0.0734	0.0786	0.0868
105	4	No	0.0374	0.0592	0.0652	0.0625	0.0721	0.0756
114	3	No	0.0190	0.0377	0.0563	0.0456	0.0589	0.0615
120	5	No	0.0580	0.0731	0.0804	0.0777	0.0853	0.0868
124	4	No	0.0369	0.0587	0.0698	0.0652	0.0760	0.0819
125	11	Yes	0.0287	0.0625	0.0724	0.0702	0.0781	0.1002
135	3	No	0.0865	0.0886	0.0907	0.0902	0.0921	0.0935
146	3	No	0.0887	0.0923	0.0959	0.0941	0.0968	0.0977
149	3	No	0.0787	0.0806	0.0824	0.0835	0.0860	0.0895
151	6	No	0.0720	0.0829	0.0929	0.0909	0.0965	0.1077
153	4	No	0.0494	0.0587	0.0775	0.0776	0.0989	0.1028
162	11	Yes	0.0535	0.0970	0.1038	0.1034	0.1172	0.1305
167	3	No	0.0432	0.0443	0.0454	0.0484	0.0511	0.0567
169	3	No	0.0245	0.0432	0.0618	0.0509	0.0641	0.0664
172	6	No	0.0523	0.0594	0.0635	0.0718	0.0869	0.1032

**Figure 6:**
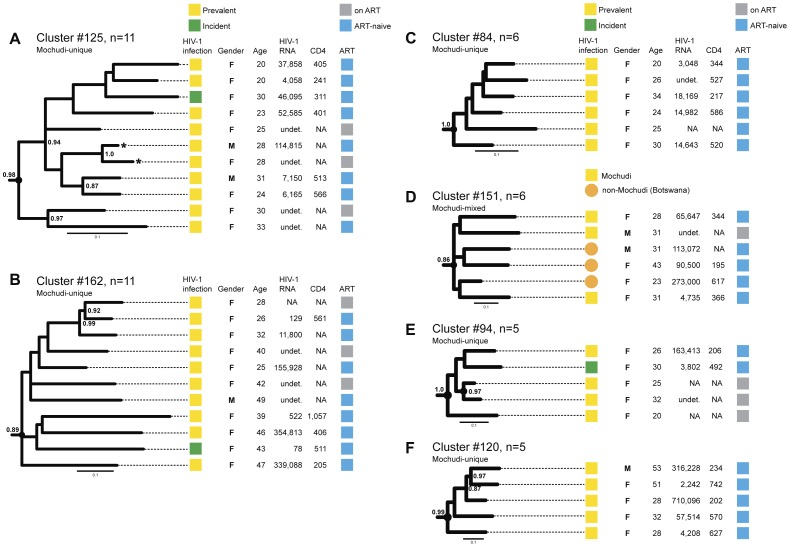
Examples of individual clusters with 3+ members. The relevant patient-specific data (prevalent or incident HIV infection, gender, age, HIV-1 RNA, CD4 count and ART status) are connected by dotted lines with the tree branches corresponding to genotyped subjects. Note: Subjects reported receiving ART through the National ARV program during the household surveys. **A:** Mochudi-unique cluster #125 with 11 members. Subjects cohabited in the same household are indicated with asterisks. **B:** Mochudi-unique cluster #162 with 11 members. **C:** Mochudi-unique cluster #84 with 6 members. **D:** Mochudi-mixed cluster#151 with 6 members. Viral sequences from Mochudi are shown as yellow squares, while sequences from outside of Mochudi are denoted by orange circles. **E:** Mochudi-unique cluster #94 with 5 members. **F:** Mochudi unique cluster #120 with 5 members.

Figure 6 depicts some of the identified clusters in the ML_PhyML_ analysis with basic demographic and clinical data (HIV-1 RNA load, CD4 count and ART-status). The Mochudi-unique cluster #125 with 11 members (Figure 6A) contained predominantly young females with one incident infection, a broad range of HIV-1 RNA load, CD4 counts between the mid-200s and mid-500s, and 3 individuals on ART. This cluster had a high bootstrap support, 98%, and demonstrated additional internal structure evident from high bootstrap support of splits within the cluster. A couple of individuals in the middle of the tree shown with asterisks cohabited in the same household. The seroconversion of incident infection in this cluster was determined over 467 days as the time between the last HIV-negative and the first HIV-positive test, which might partially explain the CD4 decline to 311 in this subject. It is possible that this seroconverter had an extended high viremia [76], as her HIV-1 RNA at the time of first positive HIV test was 4.81 log_10_ copies/ml and could be associated with rapid decline of CD4 counts in the early stage of HIV-1C infection. The HIV cluster is suggesting that a particular HIV variant has been spreading locally through a chain of viral transmissions. Short terminal branches and low pairwise distances between members of the cluster suggest they represent more recent transmissions, or are more epidemiologically related without any indication of the directionality of viral transmission. In contrast, the extended terminal branches and elevated pairwise distances within the cluster are likely to be associated with relatively old HIV transmission and/or a lesser epidemiological relatedness. Given the relatively recent HIV infection of the seroconverter in cluster #125, the observed branching topology and pairwise distances with other members of this cluster suggest that the lack of internal clustering of the incidence sequence with any other sequence within the transmission cluster #125 indicates missing data, e.g., missing index case for the identified seroconverter. In part, this can be explained by partial sampling.

Another Mochudi-unique cluster, #162 with 11 members (Figure 6B), was also predominantly female with a broader age range, also included one incident case as well as 3 ART-experienced individuals, and had less pronounced internal structure. Figures 6C to 6F illustrate phylogenetic structure combined with demographic and clinical data of another four clusters with 5 and 6 members, including one mixed cluster, #151, with sequences from other parts of Botswana. The data presented on HIV-1 RNA and CD4 count highlight individuals eligible for ART due to high levels of viral load.

Within the clustered Mochudi sequences, only 8 (4 pairs, 3.8%, 95% CI 1.8% to 7.6%) sequences originated from the same household, suggesting a broad and complex transmission network.

There was no difference in proportion of clustered sequences between subsets of ART-naïve and ART-experienced individuals from Mochudi (data not shown). Similarly, no difference in proportions of clustered sequences from Mochudi was found either within or between gender subsets (data not shown).

Among non-Botswana HIV-1C *env* sequences, 204 (16.4%; 95% 14.4% to 18.6%) clustered in 94 clusters from 2 to 5 sequences per cluster. A total of 38 (3.1%; 95% CI 2.2% to 4.2%) sequences were found in 11 clusters with 3+ members.

### HIV variants circulating in Mochudi

The branching topology of HIV-1C *env* sequences from Mochudi scattered across the phylogenetic tree suggest a diversified and mature HIV epidemic in this community. The majority of sequences from Mochudi had long terminal branches. The identified Mochudi-unique clusters, as well as non-clustered Mochudi sequences, were spread across the tree, suggesting multiple introductions of HIV to the community.

To assess the circulating HIV variants in Mochudi, clusters with 3+ members and the dyads were enumerated. Eighty-three clusters, including 22 clusters with 3+ members, with sequences from Mochudi were interpreted as at least 83 circulating HIV-1C variants circulating in the local HIV/AIDS epidemic in this community. Of those, 73 circulating viral variants were Mochudi-unique (clusters contained sequences from Mochudi only), while 10 HIV-1C variants circulate across Botswana (clusters included sequences originating from Mochudi and from other parts of the country).

**Figure 7:**
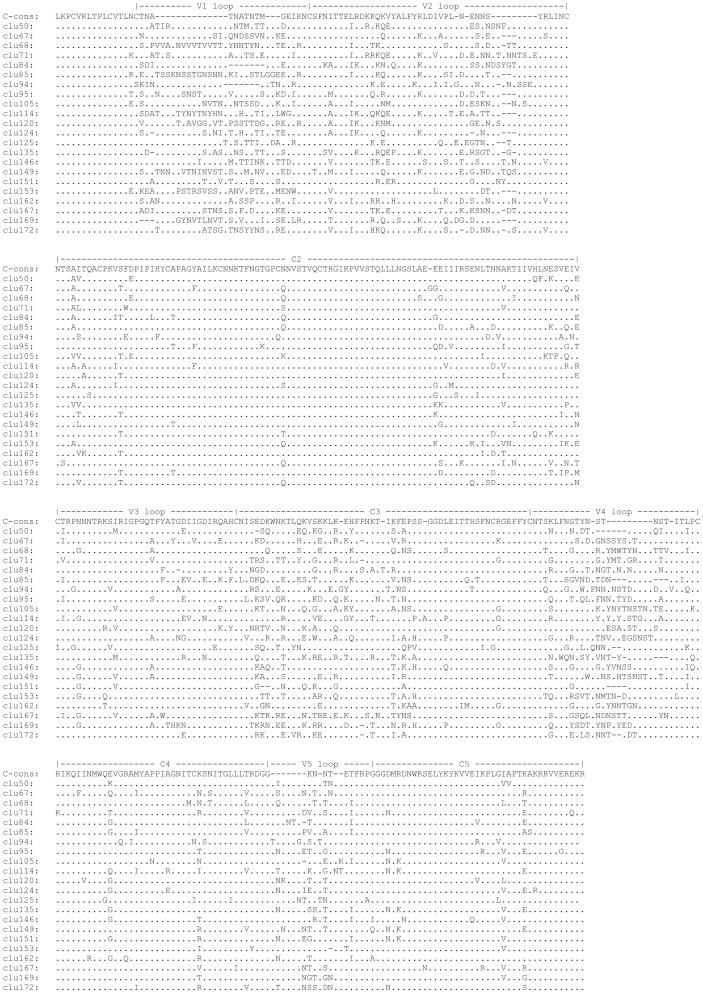
The HIV-1C *env* gp120 V1–C5 signature sequences across 22 clusters with 3+ members, VESPA analysis.

To estimate the difference between HIV variants circulating in Mochudi and identify sequence signatures, the analysis of cluster-specific sequence signatures was performed using translated amino acid alignment and VESPA (Viral Epidemiology Signature Pattern Analysis) software [77]. The sequence signatures in 22 clusters with 3+ members were analyzed against 56 randomly selected non-Botswana HIV-1C *env* sequences complemented by four HIV-1C reference sequences from the LANL HIV Database and HIV-1C consensus sequence. The VESPA analysis identified the cluster-specific amino acid signatures scattered over the V1-C5 region of the HIV-1C gp120 (Figure 7). As expected, variable loops V1, V2, V4, and V5 showed the highest density of sequence signatures. The C3 region also demonstrated a relatively high number of cluster-specific signatures. The median (IQR) of identified amino acid signatures over the V1-C5 region of gp120 was 63 (55 to 69) per cluster, ranging from 36 to 81.

### Viral clustering and HIV-1C RNA load

**Figure 8:**
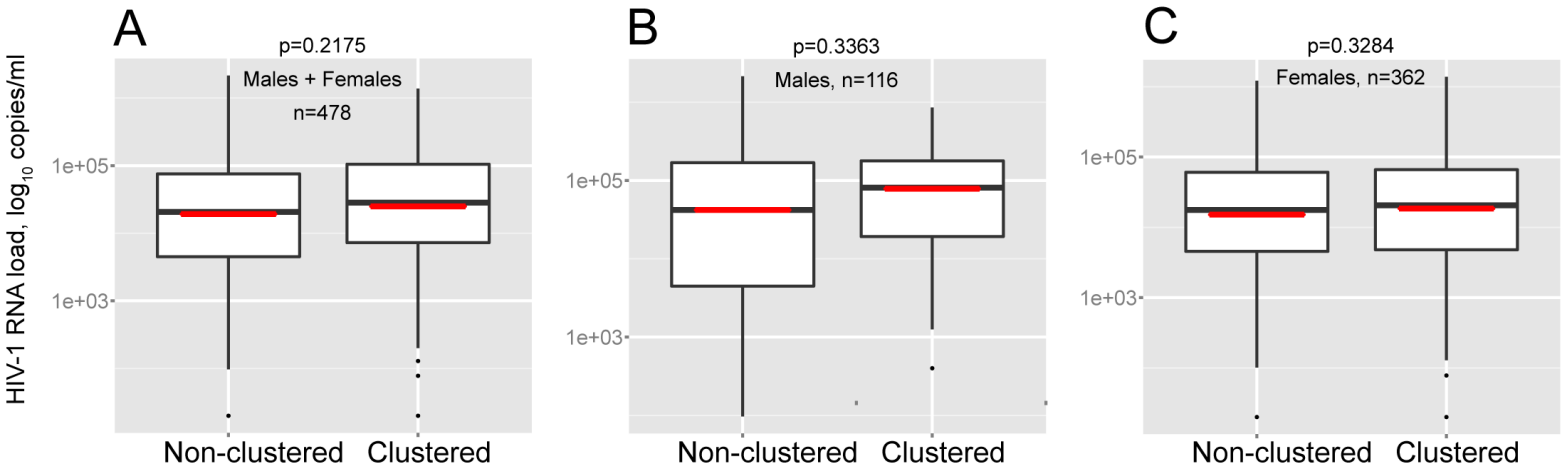
Comparison of HIV-1 RNA load within clustered and non-clustered sequences in ART-naïve individuals. The estimated p-values by Wilcoxon test are shown above each graph. **A:** The entire set of ART-naïve individuals. **B:** ART-naïve males. **C:** ART-naïve females.

The potential associations between clustering of HIV-1C *env* sequences and levels of HIV-1 RNA load were assessed. The comparison of the HIV-1 RNA levels between groups of clustered and non-clustered sequences was limited to viral sequences from Mochudi. HIV-1 RNA measurements were available for 665 subjects from Mochudi, including 181 of 212 (85.4%) clustered and 484 of 574 (84.3%) non-clustered individuals. If subjects had multiple measurements, the average value of HIV-1 RNA was used. The analysis included only ART-naïve individuals. The difference in HIV-1 RNA load between clustered and non-clustered individuals did not reach statistical significance, median (IQR) of 24,980 (4,943 to 102,800) in clustered vs. 19,200 (3,564 to 75,890) in non-clustered individuals; p = n/s (Figure 8A). No statistically significant difference in HIV-1 RNA load was observed between subsets of clustered and non-clustered men (Figures 8B) or women (Figure 8C).

### Clustering of Mochudi seroconverters

A total of 27 seroconverters were identified in Mochudi by May 2013. HIV-1C *env* sequences were obtained for 20 seroconverters. Seven of the 20 genotyped seroconverters were found in 7 Mochudi-unique clusters, with the cluster size ranging from 2 to 11 members per cluster. None of the Mochudi seroconverters clustered with another identified seroconverter, suggesting a series of independent HIV transmission events. Clusters with Mochudi seroconverters had high splits support (e.g., the bootstrap support in the ML_PhyML_ analysis was 100% in 5 clusters, 98% in one cluster, and 89% in one cluster).

The median (IQR) estimated time between the last HIV-negative and first HIV-positive tests was 474 (448 to 681) days, suggesting that, based on the midpoint estimate, most identified seroconverters were infected for about 6–12 months at the time of the first HIV-positive test. The CD4 count was quantified in 17 of 27 seroconverters and the median (IQR) CD4 count at the time of the first HIV-positive test was 385 (311 to 430). The HIV-1 RNA was measured in 20 of 27 seroconverters. Three subjects had undetectable levels of HIV-1 RNA, while median (IQR) in the remaining 17 subjects was 15,160 (5,500 to 54,520) copies/ml. Although median HIV-1 RNA load was higher in clustered seroconverters than in non-clustered (47,772 vs. 12,465 copies/ml), the difference did not reach statistical significance, likely due to a broad distribution of HIV-1 RNA within groups (IQR 14,375 to 53,249 copies/ml and 971 to 28,184 copies/ml for clustered and non-clustered seroconverters, respectively). No age difference was found between clustered (median 30, IQR 25–31 years old) and non-clustered (median 31, IQR 26–38 years old) seroconverters.

**Figure 9:**
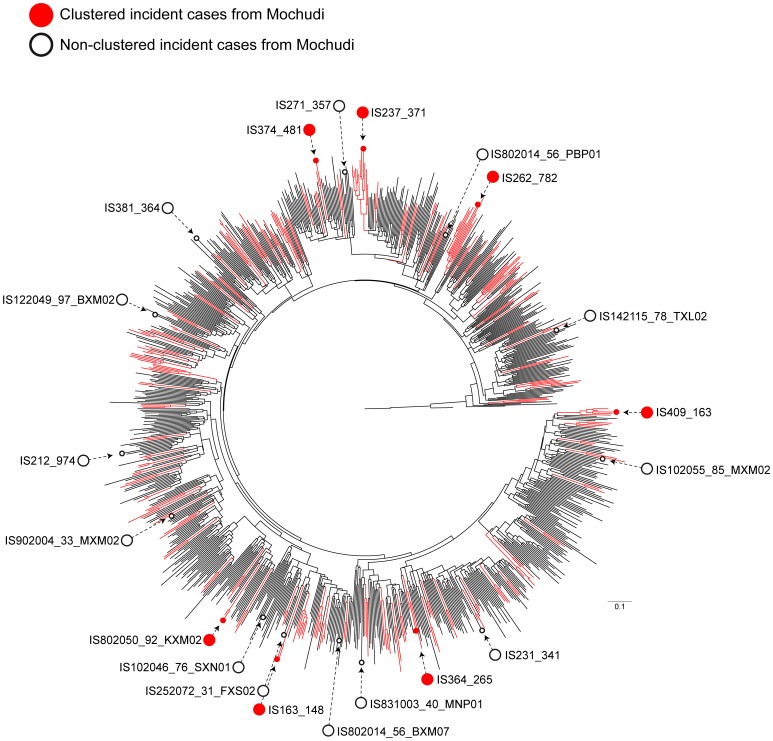
Phylogenetic relationships between Mochudi seroconverters. The tree was generated by ML_PhyML_ with 968 HIV-1C *env* sequences from Botswana. Clustered sequences with bootstrap support of splits ≥80% are shown in red. The incident HIV-1C cases from Mochudi are highlighted by circles: clustered seroconverters are indicated with filled red circles, while non-clustered seroconverters are shown with open circles.

Given that none of the Mochudi seroconverters clustered with the available HIV-1C *env* sequences from outside of Botswana, we estimated the phylogenetic relationships among Botswana sequences only, n = 968, by ML_PhyML_ inference without pre-filtering with ML_FastTree2_ (Figure 9). This analysis confirmed that identified seroconverters from Mochudi (both clustered and non-clustered) scattered across the phylogenetic tree, and none of the seroconverters clustered with another seroconverter, suggesting independent transmission chains.

## Discussion

The study presents initial results of HIV genotyping in Mochudi, a village in Botswana that is one of thousands of communities with the overwhelming burden of HIV-1C infection over recent decades. Utilizing a high sampling coverage achieved during household HIV testing and counseling campaigns in Mochudi, the study results revealed the basic structure of the local HIV transmission network by estimating and enumerating HIV-1C *env* V1–C5 clusters among genotyped viral sequences. The generated sequencing data were analyzed in the context of HIV-1C *env* sequences from other communities and countries available in the public domain.

In this study, the overall proportion of clustered HIV-1C *env* sequences was 19.1%, and was significantly higher in the subset of sequences from Mochudi as compared with the non-Mochudi sequences, 27.0% vs. 14.7%. The observed difference between proportions of clustered sequences from Mochudi and non-Mochudi highlights the importance of high sampling coverage for HIV genotyping analysis, and suggests a potential link between the depth of sampling and proportion of estimated HIV clusters in communities.

As expected, the cluster size distribution uniformly followed the power law. Similarly to the proportion of all clustered sequences, the proportion of Mochudi sequences within large clusters (3+ members) was significantly higher than the proportion of non-Mochudi sequences, 12.1% vs. 3.0%. This observation provides additional evidence for a potential association between sampling and clustering.

The analysis of Mochudi-unique and mixed clusters provides new knowledge on the structure of the local HIV transmission network in the community. The identified high proportion of Mochudi-unique clusters, 88% of all clusters with Mochudi sequences, might decrease if more HIV sequences from neighboring communities were available to contribute to the analysis. Scarce current sampling from outside of Mochudi is a limitation of this study. However, it highlights the substantial local spread of HIV in this community, justifying treatment-as-prevention as a promising public health intervention in similar settings. Surprisingly, none of the Mochudi sequences clustered with any of the 1,244 HIV-1C non-Botswana sequences. Given the substantial proportion of non-clustered Mochudi sequences, a lack of clustering between Mochudi sequences and sequences originating from outside of Botswana might point to some biased, or at least sparse or selected, sampling in the sequence sets deposited in the public database.

The genotyping coverage expressed as a ratio of available HIV-1C *env* sequences in the public database to the estimated number of HIV-positive individuals per country provided by UNAIDS (http://www.unaids.org/en/regionscountries/countries/) was 0.003% for India, 0.01% for South Africa and Zambia, 0.02% for Malawi, and 0.06% for Botswana. The topology of mother–infant pairs from two recent studies in Malawi [78], [79] highlighted in Figure 4 illustrates the point that this type of paired sequences can be used as a control for clustering. It is likely that HIV sequences in the public database represent a limited number of studies, and therefore, these sequences represent some geographic areas rather than the entire country. In contrast, the estimated genotyping coverage for Mochudi in the current study was 44% due to high sampling coverage. Therefore, it should not be surprising that the proportions of clustered HIV-1C *env* sequences in most analyses in this study were higher in Mochudi than among sequences originating elsewhere.

The following assumptions provided the rationale for enumeration of HIV variants circulating in Mochudi and identification of their sequence signatures. The ultimate goal of cluster analysis is a proper interpretation of identified clusters and viral sequences within the clusters in the context of their sampling origin. The phylogenetic clusters represent viral transmission chains, and are associated with the spread of distinct HIV variants. The cluster composition reveals the structure of a particular HIV transmission chain. Clustered viral sequences sampled from the same community provide evidence for a local spread of the specific HIV variant in this community. If sampling coverage is high, the non-clustered sequences from recently infected individuals indicate HIV infections that are likely to be acquired from an outside source.

The cluster analysis in this study identified at least 83 HIV-1C variants circulating in the local HIV/AIDS epidemic in Mochudi. Although the genotyping coverage was relatively high, it was incomplete, suggesting a larger true number of circulating viral variants in this community. The vast majority of the identified clustered HIV-1C variants, 73 of 83, were classified as Mochudi-unique, as these clusters included sequences originating from Mochudi only. In contrast to the overall estimation of the number of circulating viral variants, the estimated proportion of the Mochudi-unique HIV-1C variants is likely to be overestimated due to a sparse representation of viral sequences from the surrounding communities. This notion justifies the need for broad, systematic genotyping across the targeted area accompanied by collection of relevant socio-demographic and behavioral data in order to obtain realistic estimates of the community-unique and mixed viral variants spreading across the local HIV transmission network and to reveal mechanisms of HIV spread through association between viral clustering and risk of HIV transmission.

Whether association between sampling/genotyping coverage and clustering is a linear function, or it follows a more complex non-linear relationship, still needs to be addressed. Assuming a linear dependency, we can estimate the true proportion of clustered HIV-1C *env* sequences in Mochudi as 0.61 (0.27/0.44), which suggests that about 61% of all HIV-positive individuals in this community should be in clusters. The true proportion could be higher, if the association between sampling and clustering is not linear.

A potential association between viral clustering and HIV-1 RNA load is of particular interest, as it provides the most direct link to the spread of a particular viral variant. The difference in HIV-1 RNA levels between clustered and non-clustered ART-naïve individuals did not reach statistical significance. The presence of old HIV infections in both subsets of clustered and non-clustered individuals could probably explain the observed lack of significant difference. This observation highlights the importance of focusing the cluster analysis primarily on recently transmitted HIV infections.

Clustering patterns of new incident HIV cases, or seroconverters, is critical for better understanding the underlying mechanisms and dynamics of HIV transmission network. In this study no clustering was observed among seroconverters, and 7 out of 20 genotyped seroconverters were found in the Mochudi-unique clusters. This translates into 35% with a broad 95% CI from 16% to 59%. Given the 44% genotyping coverage in Mochudi, and assuming that sampling and genotyping coverage are equal among prevalent and incident cases, the predicted proportion of clustered seroconverters is about 19.4% (0.44 in prevalent cases * 0.44 in incident cases), which is at the lower end of the actual 95% CI. Seroconverters in this study were identified prospectively by a combination of HIV-negative and HIV-positive tests during household surveys. The median of HIV-1 RNA in seroconverters at the time of the first positive HIV test was relatively low suggesting that most of seroconverters were identified rather at the end of their primary HIV infection. The CD4 levels in most Mochudi seroconverters were relatively low but did not differ from CD4 levels in the prospective cohort of individuals with primary HIV-1C infection and estimated time of seroconversion in our recent study in Botswana [76], [80] (Table 5). This is evident from comparison of CD4 levels in Mochudi seroconverters with those in participants in the prospective cohort at time intervals 100 to 300 days post-seroconversion (p/s; p-value = 0.33), 200 to 400 days p/s (p-value = 0.88) and 400 to 800 days p/s (p-value = 0.67; all tests are Wilcoxon rank sum test). Seroconverters in the prospective cohort [76], [80], [81] were followed up monthly and initiated ART as soon as their CD4 dropped below the National Guidelines threshold. Ten of 14 extended high viremics from the prospective cohort started ART within the first 500 days p/s due to reduced CD4 counts, and only pre-ART assessments of HIV-1 RNA and CD4 counts were included in the analysis in the prospective cohort [76], [80], [81]. Given that in this study the median (IQR) estimated time from HIV transmission to identification of seroconverters was 474 (448 to 681) days, the observed low CD4 counts in seroconverters at the time of the first positive HIV test should not be surprising.

**Table 5:** CD4 levels in the prospective cohort of individuals with primary HIV-1C infection in Botswana and seroconveters in this study.

	n	Min	1^st^ quartile	Median	Mean	3^rd^ quartile	Max
Prospective cohort							
Less than 50 days p/s*	27	242	292	461	487	586	1,357
100–200 days p/s*	42	167	299	448	490	578	1,378
100–300 days p/s*	42	167	291	431	481	570	1,437
200–400 days p/s*	37	169	277	387	460	529	1,394
400–800 days p/s*	29	200	324	381	470	542	1,383
Seroconverters in this study	19	70	312	385	429	461	1,334

post-seroconversion, p/s.

The results of cluster analysis should not be taken as final because genotyping of the collected samples in Mochudi is still a work in progress, and 7 recently identified seroconverters have not been genotyped yet. We believe that increased genotyping coverage for both prevalent and incident HIV-1C infections from Mochudi will provide more accurate estimates of clustering patterns of incident HIV-1C cases in this community.

The non-clustered sequences indicate a substantial presence of missing data and represent unknown, or hidden, chains of HIV transmission. At least three possible sources/reasons may contribute to and explain the presence of non-clustered sequences: (1) un-sampled HIV-infected individuals from the local community, particularly men, (2) individuals who moved out of the community or are deceased, and (3) HIV transmissions from an outside source.

Given the predominance of heterosexual mode of HIV transmission in Botswana, the skewed gender composition of identified clusters toward women indicates under-sampling of men in this community. In fact, despite the high overall sampling coverage in Mochudi during household surveys, the gender ratio, males:females, was 1∶3, pointing to a substantially lower rate of male participation. This is also consistent with the predominance of women among identified seroconverters in Mochudi. The skewed gender participation in household surveys in Mochudi suggests existence of barriers preventing men from accessing HIV prevention, treatment and care apparently due to fear of stigma, or losing a job, or being perceived as “weak” or “unmanly”. It seems likely that an increase in male participation could resolve at least some of the non-clustered incident cases, which requires dedicated research to identify true challenges and opportunities. Apparently, novel strategies aiming to target couple testing, or identify “hard-to-reach” populations in community (focusing primarily on men) need to be developed and implemented. Other studies on HIV/AIDS in Botswana open to individuals of both genders have also found that participation by females is higher. [82], [83]

At least some fraction of the non-clustered viral sequences are likely to indicate an outside source of HIV transmission. This is particularly critical for new incident infections, as they represent the most recent dynamics in a local HIV epidemic. The proportion of HIV transmissions originating from an outside source including adjacent communities is critical for optimization of prevention interventions and proper interpretation of results from combination prevention studies. The village of Mochudi is located relatively close to the national capital, Gaborone. It is likely that such a close geographic proximity facilitates frequent job-related and recreational travel, and may be associated with mixed sexual networks and HIV transmissions originating from Gaborone. Although the study in Mochudi had no power to assess the outside sources of HIV transmission, the results of the study clearly show the importance of a broader picture of HIV transmission networks.

Additional factors contributing to the existence of non-clustered sequences could be sampling specifics of the HIV-1C *env* sequences deposited in the public database, and intra-host evolution of HIV. Sparse sampling is one of the study limitations which could be responsible for missing links between HIV-1C *env* sequences from Mochudi and viral sequences originating from outside of Botswana. The impact of intra-host evolution on phylogenetic clusters could be substantial because clusters identified in Mochudi represent HIV transmission chains accumulated over time, and the majority of analyzed cases were prevalent with unknown time of HIV infection. Given high evolutionary rates within the analyzed HIV-1C *env* gp120 V1-C5 region (approximately 3 times higher than in HIV-1C *gag* [47]) viral sequences sampled during chronic infection are likely to be highly diversified, and therefore are less likely to form clusters. This highlights the importance of early sampling during the time period that is close to the HIV transmission event.

The presented analysis is based on sequencing of the *env* gp120 V1-C5, a highly divergent region of the HIV-1 genome. It would be important to compare different structural and non-structural HIV-1 genes for their performance in cluster analysis. The divergent viral genes with high intra-host evolutionary rates, such as *env* gp120, might be advantageous in cluster analysis of HIV infections sampled close to the time of viral transmission. In contrast, more conserved viral genes with relatively low intra-host evolutionary rates, such as *gag* or *pol*, might be more informative for analysis of chronic HIV infections with unknown/long time of HIV transmission. However, as most analyzed data sets include both recent and old HIV infections, the ideal data set might need to include multiple HIV genes, or get as close to near full-length viral sequencing as possible.

In summary, to assess the basic structure of the HIV transmission network in a community, we performed an HIV genotyping study in the village of Mochudi, Botswana. The results of the study highlight the importance of high sampling coverage, early sampling, and targeting both prevalent and incident cases in communities. The high proportion of Mochudi-unique clusters among clustered sequences suggests that the HIV epidemic in communities is dominated by locally circulating viral variants, although this notion needs to be confirmed or refuted by genotyping data from other neighboring and remote communities. We suggest a two-step approach for understanding and interpretation of the HIV genotyping data in communities. We conclude that mapping of circulating HIV variants in communities is a critical step toward better understanding of the structure of local HIV transmission networks. The map of circulating HIV variants could be generated by genotyping of prevalent HIV infections sampled in the targeted communities, which provides important baseline information and is absolutely essential for analysis of HIV spread across communities. In the next step we propose to focus on sampling and HIV genotyping of incident HIV cases in the same communities. Combined with the map of circulating HIV variants, the genotyping data on incident HIV cases should have great power to reveal the dynamics of the local HIV transmission networks. The results of this analysis have the potential to optimize HIV prevention strategies aimed at stopping the spread of HIV across communities.

## Supporting Information

Table S1 Accession numbers.
(DOCX)

Table S2 Recombination analysis of HIV-1C *env* gp120 V1–C5 sequences from Botswana by RPD4: the Bonferroni-corrected p-values are shown for RDP, GENECONV, BootScan, MaxChi, Chimaera, SiScan, PhylPro, LARD, and 3Seq.
(XLSX)
