# Supplementary material for: Phylogenetic Relatedness of Circulating HIV-1C Variants in Mochudi, Botswana
Source: PLoS One. 2013 Dec 11;8(12):e80589. doi: 10.1371/journal.pone.0080589 (PMC3859477; doi:10.1371/journal.pone.0080589)
Supplement: Table S1 — Accession numbers. (DOCX) [file pone.0080589.s001.docx]

Supplementary Table 1. Recombination analysis of HIV-1C *env* gp120 V1-C5 sequences from Botswana by RPD4: the Bonferroni-corrected p-values are shown for RDP, GENECONV, BootScan, MaxChi, Chimaera, SiScan, PhylPro, LARD, and 3Seq.

| Sequence ID | Accession number | Major parent | Minor Parent | RDP | GENECONV | BootScan | MaxChi | Chimaera | SiScan | PhylPro | LARD | 3Seq |
| --- | --- | --- | --- | --- | --- | --- | --- | --- | --- | --- | --- | --- |
| S152110_82_MVM01 | KF374235 | S416_576 | Unknown | 4.68x10^-2^ | 2.71x10^-3^ | 7.63x10^-5^ | – | – | 2.16x10^-4^ | – | – | – |
| S372203_64_NXM01 | KF374489 | S402005_99_SXK03 | S218_156_1 | – | 6.65x10^-8^ | 6.40x10^-4^ | 1.06x10^-2^ | – | – | – | – | – |
| S802029_71_OOP01 | KF374631 | BW_TH874640 | 00BW18802 | – | – | – | 2.2x10^-2^ | 4.27x10^-2^ | – | – | – | – |
| BW_TH894780 | KF373854 | S144_887 | Unknown | – | 5.77x10^-4^ | – | – | – | 1.11x10^-31^ | – | – | – |
| 96BW1104 | AF110969 | BW_TH873531 | Unknown | 1.14x10^-2^ | 4.8x10^-3^ | 2.47x10^-3^ | – | – | – | – | – | – |
